# Dietary Response of Black‐Backed Jackals (*Lupulella mesomelas*) to Contrasted Land Use

**DOI:** 10.1002/ece3.72186

**Published:** 2025-10-09

**Authors:** Megan Roberts, Fanny Degrugillier, Locadia Dzingwena, Hervé Fritz, Virginie Rougeron, Franck R. Prugnolle

**Affiliations:** ^1^ International Research Laboratory, REHABS, CNRS‐NMU‐UCBL George Campus, Nelson Mandela University George South Africa; ^2^ Infectious Diseases and Vectors: Ecology, Genetics, Evolution and Control (MiVEGEC) University of Montpellier, CNRS, IRD Montpellier France; ^3^ Sustainability Research Unit, George Campus Nelson Mandela University George South Africa

**Keywords:** blacked‐back jackal, DNA metabarcoding, flexible diet, scat analysis

## Abstract

Black‐backed jackals are medium‐sized canids with an omnivorous and opportunistic diet, allowing them to persist across different landscapes and land uses in southern Africa. Their diet is influenced by both top‐down factors, like the presence of larger carnivores, and bottom‐up factors, such as prey size, abundance, behavior, and habitat. Therefore, varying land uses—such as livestock farming, game ranching, or nature reserves—can significantly impact jackal diet and behavior due to variations in management strategies and species presence. We examined the dietary response of jackals to three land‐use types in South Africa: a nature reserve with larger carnivores, a game farm without larger carnivores, and livestock farms in the semi‐arid Karoo, South Africa. Using DNA metabarcoding of jackal scats, we identified prey species across these landscapes. Results showed significant variation in diet, with the jackals in the nature reserve displaying a broad diet, while livestock and game farms' diet was dominated by specific prey species, such as sheep on livestock farms and greater kudu on the game farm. Rodents were a significant dietary component for jackals across all land uses, highlighting their importance as a resource. Similarly, large ungulates (greater than 100 kg) consistently comprised a stable part of the jackal diet across different land uses. Seasonal shifts indicated that rodents served as a crucial resource during the dry season, while medium‐sized ungulates saw increased consumption during the wet season. Notably, on the livestock farm, the consumption of steenbok peaked during the autumn and summer collection periods, even surpassing that of sheep. This study highlights the behavioral flexibility of jackals, illustrating their ability to adapt their diets based on prey availability, habitat conditions, and the presence of other predators.

## Graphical abstract

This study examines dietary shifts in black‐backed jackals across different land‐use types using DNA metabarcoding. Results reveal that jackal diets vary significantly by landscape and season, with a strong reliance on sheep in livestock areas, selective feeding on greater kudu on game farms, and a broader, more even prey profile in nature reserves. Detection of DNA from other carnivores suggests potential scavenging and interspecies interactions.

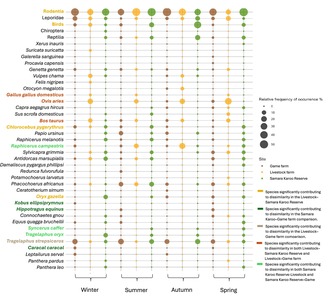

## 1 Introduction

**FIGURE 1:**
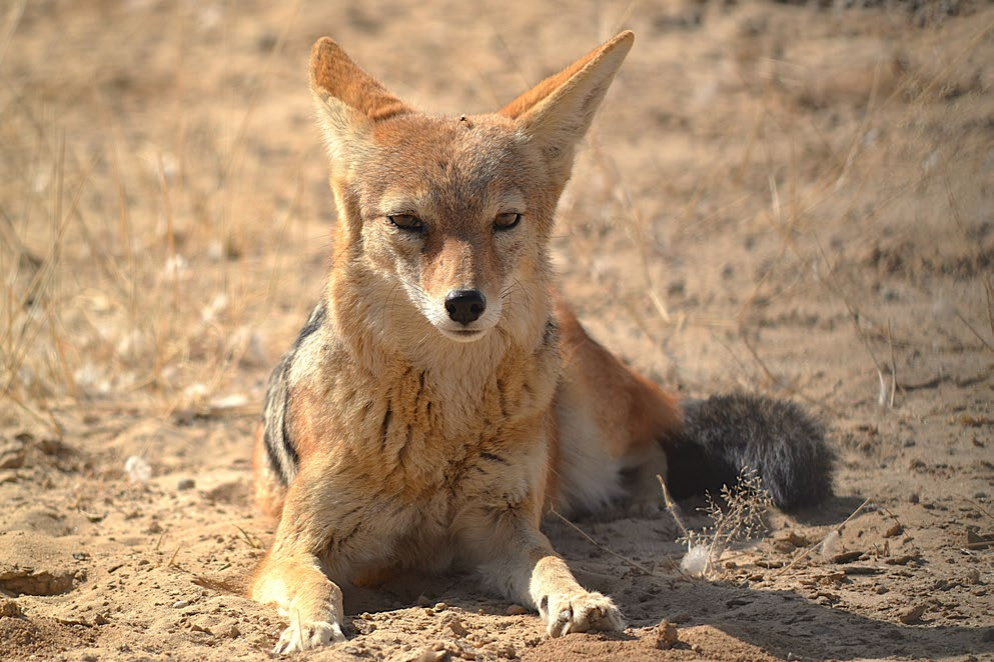
Black‐backed jackal (*Lupulella mesomelas*). Photograph taken by M. Roberts.

Black‐backed jackals (*Lupulella mesomelas*; Figure 1) face very few detectable barriers when dispersing across South Africa and can cover large distances without being hindered by fences (Bothma 1971a; Ferguson et al. 1983; Humphries et al. 2016; Minnie, Avenant, et al. 2018; Minnie, Zalewski, et al. 2018). Despite centuries of population control efforts, black‐backed jackals thrive on farmlands and in most conservation areas across South Africa (Minnie, Avenant, et al. 2016). Given the black‐backed jackal's wide habitat tolerance, with high reproductive output and dietary flexibility, this species remains a common inhabitant in changing anthropogenic landscapes (Fourie et al. 2015; Hayward et al. 2017; Minnie, Gaylard, and Kerley 2016; Kerley et al. 2017; Minnie, Zalewski, et al. 2018; Kamler et al. 2020).

Historically, black‐backed jackals were predominantly viewed as scavengers, relying heavily on resources provided by larger carnivores (Smithers 1971; Rowe‐Rowe 1975; Stuart 1976; Loveridge and Macdonald 2003; Skinner and Chimimba 2005). However, the removal of many large carnivores over a century ago, particularly in areas dedicated to agricultural practices and livestock production, has resulted in smaller carnivores like the black‐backed jackal becoming top predators in these ecosystems (van Sittert 2016). This shift indicates that jackals are not solely dependent on large carcasses for their diet; instead, they have adapted to exploit a broader range of prey resources. This has created the opportunity for black‐backed jackals to expand their ecological niche (Rowe‐Rowe 1992; Skinner and Chimimba 2005; Klare et al. 2010; Minnie, Avenant, et al. 2016; Kerley et al. 2017; Minnie, Zalewski, et al. 2018).

Studying the diet of black‐backed jackals is crucial for understanding their success and adaptability in colonizing a wide range of habitats, including human‐altered landscapes, which can provide valuable insights for wildlife management and conservation (Minnie, Avenant, et al. 2016; Minnie, Zalewski, et al. 2018; Drouilly et al. 2018; Woodgate et al. 2023). Carnivore resource selection is primarily influenced by two key factors: prey abundance and landscape features that affect prey availability and vulnerability (Stephens and Krebs 1986; Balme et al. 2007). Assessing jackal diets can provide valuable insights into the prey species they impact, their competition with or facilitation from other predators, their dietary flexibility, and their reliance on specific prey. Moreover, examining species interactions in the context of human impacts offers important perspectives on species adaptation, community dynamics, and ecosystem stability.

Black‐backed jackals, as omnivores, exploit a broad range of food items depending on availability, from opportunistic scavenging to actively hunting prey, including young fawns hidden in vegetation (Rowe‐Rowe 1982; Loveridge and Macdonald 2003; Van de Ven et al. 2013; Hayward et al. 2017). Observations show that jackals are capable of hunting ungulate species exceeding 25 kg, such as bushbuck (*Tragelaphus sylvaticus*), springbok ( *Antidorcas marsupialis* ), and impala ( *Aepyceros melampus* ), demonstrating that they do not solely rely on scavenging from larger predators to access prey of this size (Van der Merwe et al. 2009; Klare et al. 2010; Brassine and Parker 2012; Yarnell et al. 2013; Hayward et al. 2017).

Black‐backed jackals exhibit dietary flexibility, adjusting their intake of small and large mammals based on resource availability. In the presence of larger carnivores, they may rely more heavily on carrion (Fourie et al. 2015; Roberts et al. 2024), whereas in areas without larger carnivores, they actively hunt smaller, more accessible prey—including livestock, particularly in sheep‐dominated farming regions (Klare et al. 2010; Kamler, Stenkewitz, et al. 2012; Fourie et al. 2015). Land‐use practices, such as livestock and game farming, agriculture, and conservation, further influence jackal diets. In livestock areas, they may scavenge or prey on domestic animals, often leading to conflict with farmers (Jansen 2016; Drouilly et al. 2018). Agricultural zones offer rodents and other small prey near crop fields (Humphries et al. 2016), while game farms and conservation areas provide wild prey, whose availability depends on local management strategies.

Thus, jackal diets are shaped by both top‐down forces, like the presence of larger carnivores, and bottom‐up factors, including prey size, abundance, behavior, and habitat (Fuller and Sievert 2001; Hayward et al. 2017; Nattrass et al. 2019). These ecological and anthropogenic variables drive regional differences in feeding behavior, complicating broad generalizations (Tambling et al. 2018; Nattrass et al. 2019). Recent studies have begun addressing these complexities, particularly by comparing jackal diets across livestock farms and protected areas to inform conflict mitigation strategies (Forbes 2011; Humphries et al. 2016; Jansen 2016; Drouilly et al. 2018; Pardo et al. 2022). Given their adaptability, it is essential to incorporate current data on jackal feeding ecology when managing changing human‐dominated landscapes (Codron et al. 2018).

Among other methods to study the diet, DNA metabarcoding offers a timely method to gather this information and has proven to be an accurate and effective method for analyzing the diets of various species using fecal samples as a source of DNA in comparison to traditional methods (Taberlet et al. 2012; Shehzad et al. 2012; Forin‐Wiart et al. 2018). The application of DNA metabarcoding has been used as an approach to overcome the biases of traditional diet analysis studies since it has been shown to be successful in studying a more complete foraging niche of predator species (Symondson 2002; Shehzad et al. 2012; Xiong et al. 2017; Forin‐Wiart et al. 2018; Lesilau 2019; Thuo et al. 2019; Beng and Corlett 2020).

In this study, we investigated the year‐round diet of black‐backed jackals across three distinct land‐use types in the Greater Karoo, South Africa, using DNA metabarcoding. We compared jackal dietary patterns in a nature reserve with lions ( *Panthera leo* ) and cheetahs ( *Acinonyx jubatus* ), a game farm without these apex predators, and adjacent livestock farms.

Our research addressed three main questions:

- How does the diet of black‐backed jackals vary across these different land‐use types, each with unique species compositions and wild ungulate populations?
- What is the overall diet of jackals in each landscape, and how does it change seasonally?
- Does the presence or absence of larger predators influence jackal consumption of large wild ungulate species, and does this scavenging impact the consumption of smaller prey species?

By addressing these questions, we aim to provide a comprehensive understanding of how black‐backed jackals adapt their foraging strategies in response to land use and predator presence. This knowledge is vital for developing effective management strategies that balance conservation objectives with agricultural interests, helping to mitigate human–wildlife conflict while supporting ecological stability in both protected and modified environments. Additionally, when ungulates were frequently detected in the diet, we considered ecological context such as seasonal patterns, ungulate body size, species presence, and birthing behavior to interpret whether their consumption was more likely due to active predation or scavenging.

## 2 Methods

### 2.1 Study Site

Our study was conducted in the Eastern Cape Province of South Africa, approximately 50 km outside Graaff‐Reinet, in an area encompassing three contrasting land‐use types: (i) farmland for small livestock and cattle, (ii) a nature reserve with large carnivores, and (iii) a game farm without large carnivores. This area, which we will refer to as the greater Samara, is part of the Great Karoo, a region with diverse habitats and containing over 60 mammal species. The greater Samara features a mosaic of vegetation types spread across four biomes: Nama‐Karoo, Savanna, Thicket, and Grassland (Van Cauter 2004). Situated on the edge of the Sneeuberg mountain range, the greater Samara landscape includes the flat plains typical of the Karoo, mountainous plateaus, and dense thicket‐filled valleys. Covering 28,300 ha of various land uses, the greater Samara has been undergoing rewilding since 1997.

**FIGURE 2:**
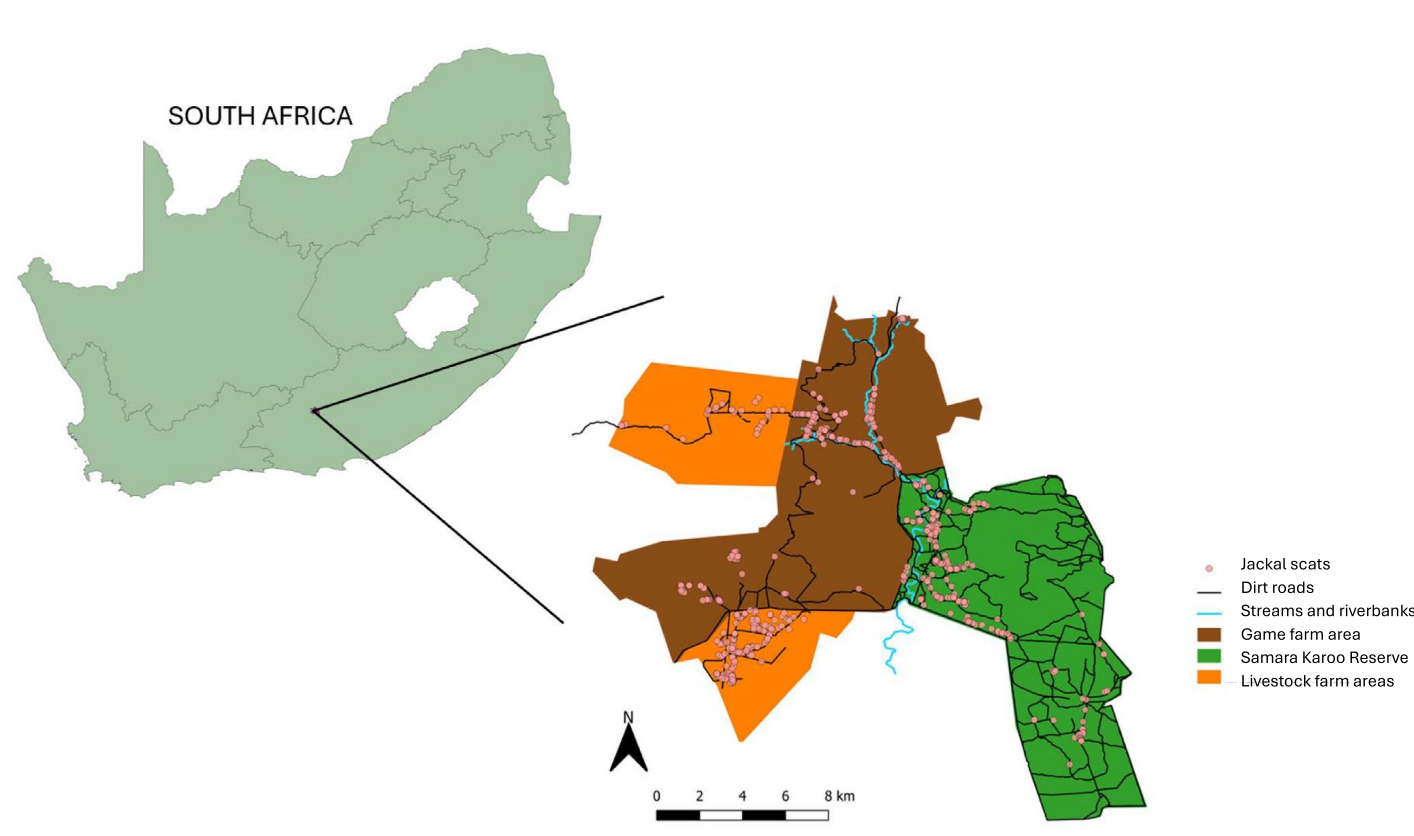
Map illustrating the three distinct land‐use types in the study area, Eastern Cape, South Africa. The orange polygons represent livestock farms, which primarily include sheep along with some cattle and Angora goats. The brown polygon denotes the game farm, where larger carnivores are not present. The green polygon marks the Samara Karoo Reserve, where larger carnivores are present.

The Samara Karoo Reserve (Figure 2), a 12,070 ha section of this area, reintroduced cheetahs in 2003 and lions in 2019. It now hosts big‐five species alongside other Karoo inhabitants like gemsbok ( *Oryx gazella* ), eland (*Tragelaphus oryx*), springbok, Cape Mountain zebra ( *Equus zebra* ), Burchell's zebra ( *Equus quagga burchellii* ), and black wildebeest ( *Connochaetes gnou* ). At the time of the study, the reserve was home to one lion pride, along with a female cheetah with cubs and a male cheetah coalition.

The Melk River, which is a tributary of the Sundays River south of Graaff‐Reinet, flows intermittently through the study area, starting in the southwest near the Samara Karoo Reserve and meandering through the game farm. During colder months, rainfall and melting snow from the mountains fill parts of the valleys with water. For our study, what we define as the game farm, covering 13,585 ha (Figure 2), is an aggregate of various properties managed under the greater Samara, and is largely dominated by areas without any livestock, with the exception of the southwestern part where cattle were rarely grazed during the whole study. The game farm supports abundant wildlife, including greater kudu ( *Tragelaphus strepsiceros* ), warthog ( *Phacochoerus aethiopicus* ), and gemsbok, along with smaller herds of blesbok (*Damaliscus pygargus phillipsi*), springbok, black wildebeest, red hartebeest (*Alcelaphus buselaphus caama*), eland, sable ( *Hippotragus niger* ), roan antelope ( *Hippotragus equinus* ), and mountain reedbuck ( *Redunca fulvorufula* ). The livestock farm land use, as we define it in this study (Figure 2), covering 7412 ha, is composed of two sections, with three different owners. The southern section is predominantly stocked with meat‐master sheep, which are the most prevalent livestock, and smaller herds of cattle. During the study period, lambs were present throughout the year in the southern section, as rams were left with the herd and breeding occurred year‐round. The northern section hosted Angora goats, sheep, and cattle, and consisted of camps belonging to farms from the Dugmore and De Lange families. Livestock farmers in this region often own small herds of game animals, including red hartebeest, springbok, and ostrich ( *Struthio camelus* ), which are present on both livestock farm sections. The northern section also has blesbok and black wildebeest. Greater kudu, which are not owned, frequently move between farms by jumping over fences, while warthogs often dig beneath fences and crawl through to access different farms. Additionally, wild species such as hares (Leporidae), steenbok ( *Raphicerus campestris* ), and common duiker ( *Sylvicapra grimmia* ) are commonly found across the farms.

### 2.2 Sample Collection and Storage Protocol

Scats were collected at four periods from May 2023 to February 2024, broadly ensuring coverage of each season. The first collection took place at the end of autumn in May, the second at the end of winter in August, the third at the end of spring in November, and the last one at the end of the summer in February. Collection was performed at each site (the Samara Karoo Reserve with larger carnivores, the game farm without larger carnivores, and the livestock area) for at least 10 days at each sampling period (Figure 2). Previous collections made by our team in other reserves indicated that this sampling effort should be sufficient to reach at least 30 jackal scats per site and sampling period, and hence a minimum total of 120 scat samples, which was suggested to be adequate to describe carnivores' diet according to previous studies (Kaunda and Skinner 2003; Trites and Joy 2005; Klare et al. 2010; Van de Ven et al. 2013; Wang et al. 2022).

Scat samples were mostly collected early in the morning, from 05:00 to 11:00, and in the late afternoon, when there was still some daylight, from 16:00 to 18:00. A combination of road transects from a vehicle and walking along wild paths was conducted across the three sites, during which road verges and surroundings were inspected for scats. We collected fresh scats when available and intact older scats, ranging from a day to 2 weeks old, as previous studies have shown sufficient DNA remains during this time (Thuo et al. 2019; Massey et al. 2021). Scat samples were collected in sterile 50 mL tubes and kept in a cooler during transport to the field station, where they were then stored in a freezer at −20°C. The sterile handling and immediate freezing of samples minimize DNA degradation and ensure the integrity of the samples for downstream molecular studies. At the end of sampling for all 4 months, scats were transported to the laboratory at Nelson Mandela University (NMU), Port Elizabeth campus, where they were stored until processed for molecular analyses.

### 2.3 DNA Extraction and PCR Amplification

The DNA of 420 feces was extracted using 180–220 mg of fecal material taken from the inner parts of the scat, since prey and predator DNA may not be evenly distributed within a scat sample (Deagle et al. 2009; Forin‐Wiart et al. 2018). DNA extraction was performed using the QIAamp DNA Stool Mini Kit (Qiagen Inc, Valencia, CA, USA), following the manufacturer's instructions. Extraction blanks, containing no fecal sample, were included in each extraction batch (every 23 samples) as a negative control to test for potential contamination (Deagle et al. 2009; Forin‐Wiart et al. 2018; Shao et al. 2021).

To identify the vertebrate species consumed by black‐backed jackals, we amplified and sequenced a fragment of the V5 loop of the mitochondrial 12S rRNA gene using degenerate 12SV5 primers (Riaz et al. 2011). These primers are conserved across vertebrates and enhance taxonomic coverage by incorporating degenerate base pairs (Riaz et al. 2011). Given that the primers also amplify jackal DNA, which is present in far greater quantities than that of the prey, we designed a blocker specific to black‐backed jackal DNA (5′‐CCACTATGCCTTAGCCCTAAACATAGATAATCCTAC‐Spacer_C3‐3′) to limit its amplification (Vestheim and Jarman 2008; Boessenkool et al. 2011; Shehzad et al. 2012; Pang 2021).

We employed a two‐step PCR to construct the amplicon libraries. The first round of PCR utilized the modified 12SVdeg5 primer sets from Riaz et al. (2011), with the following sequences: 12SV5degForward: 5′‐YRGACAAGTCCACAATCTAG‐3′ and 12SV5degReverse: 5′‐TTAGATACCCCACTATGY‐3′. In this step, the 12SVdeg5 primers incorporate unique barcodes represented by variable lengths, such as N, NN, and NNN. These barcodes are specific to each sample, allowing for their identification post‐sequencing. Additionally, 5′ nucleotide overhangs were included in these primers, resulting in a combination of six primers in our first PCR (overhang‐N‐12SV5degF, overhang‐N‐12SV5degR, overhang‐NN‐12SV5degF, overhangNN‐12SV5degR, overhang‐NNN‐12SV5degF, and overhang‐NNN‐12SV5degR), adapted from Pang (2021) (Table S1). Black‐backed jackal blockers were added during this first round of PCR. The overhangs serve to anchor the second round of PCR, which introduces indexes and completes the Illumina adapters.

The PCR conditions were performed in a total volume of 33 μL, comprising 2 U of Platinum Taq DNA Polymerase (Life Technologies, 10966034), 3.5 μL of 10 × PCR buffer, 1.75 μL of 50 mM MgCl_2_, 3.5 μL of 2.5 mM dNTPs, 0.47 μL of each combination of the six primers at concentrations of 10 μM, 13.49 μL of double‐distilled water (ddH_2_O), 50–80 ng of DNA extracts, and 2.8 μL of 25 μM jackal blocker. The PCR cycling parameters included an initial activation step of 10 min at 95°C, followed by 45 cycles consisting of 30 s at 95°C for DNA strand denaturation, 30 s at 60°C for primer annealing, and 30 s at 68°C for the blocking oligonucleotide to bind to target sequences. An elongation step was performed at 72°C for 10 min, followed by a final cooling step at 10°C for 10 min to stabilize the amplicons before sequencing. To assess amplification success and amplicon sizes, PCR products were analyzed by gel electrophoresis on a 2% agarose gel. Extraction blanks and negative PCR controls (substituting ddH_2_O for the DNA extract) were run alongside the PCR.

In the second round of PCR, the amplicons generated from the first PCR are combined with complete Illumina adapters (P5 and P7), which are essential for the sequencing process as they facilitate the binding of the fragments to the flow cell during sequencing (Ando et al. 2020). This step also introduces additional unique indexes, typically 9 bp in length, designed to further differentiate the samples and prevent index hopping by ensuring that each sample has a distinct identifier.

This process was carried out by the Genseq Platform at the University of Montpellier, France, with libraries prepared according to Illumina's two‐step PCR protocol for amplicon sequencing. The Genseq platform utilized the first PCR amplicons and conducted a second PCR with 10 cycles, adding dual index primers designed according to the following scheme:

I5 = AATGATACGGCGACCACCGAGATCTACAC(9 nt)TCGTCGGCAGCGTC and I7 = CAAGCAGAAGACGGCATACGAGAT(9 nt)GTCTCGTGGGCTCGG.

The 9 nt sequences for I5 and I7 primers were identical, creating a unique combination of indices. After the second PCR, the products were pooled and bead‐purified at a DNA ratio of 0.7:1. The final library was quantified using qPCR and then loaded onto a MiSeq Reagent Kit V3 cartridge (300 cycles) for paired‐end sequencing on a MiSeq system.

### 2.4 Bioinformatic Analyses of Sequences

The overall quality of the sequence reads was evaluated with MultiQC version 1.14, which provides a comprehensive report of quality metrics across multiple samples (Ewels et al. 2016). Sequences with an average *Q*‐score below 30 were excluded from further analyses, ensuring that low‐quality reads were excluded (Ewels et al. 2016). Paired‐end sequencing reads were filtered through a bioinformatics pipeline (provided by Illumina) to trim and sort the reads into individual FASTQ files corresponding to each scat sample. Sequence reads that were not assigned were removed (Martin 2011). Cutadapt software version 3.5 was used to trim the 12S primers from the sequence reads, to remove non‐target regions from the sequences (Martin 2011).

The trimmed forward and reverse reads of each paired‐end read were merged in silico by aligning the forward and corresponding reverse sequences using USEARCH software version 11.0.667 (Edgar 2010). Further, the merged sequences were quality‐filtered to reduce the number of sequencing errors and PCR artifacts, allowing a maximum of only one expected erroneous base per merged read, using USEARCH version 11.0.667 (Edgar 2010). Clustering of sequences was formed into operational taxonomic units (OTUs) using the UPARSE algorithm implemented in USEARCH, which effectively removes chimeras and singletons in the process (Edgar 2013). The representative sequence for an OTU is the most abundant sequence within the cluster, and all sequences in the cluster were required to share 97% similarity. The OTU sequences were taxonomically assigned using BLAST (Basic Local Alignment Search Tool) on NCBI to search for the best hits of vertebrate sequences in the GenBank database. Taxonomic assignments were assigned at the species level when an OTU matched with a single locally occurring species in the database with a “best hit” of ≥ 95% of identity or assigned to the lowest taxonomic level when an OTU matched to more than one locally occurring species with ≥ 95% identity. OTU sequences with < 95% identity to their closest match on BLAST were classified as “undetermined” and excluded from further analysis. The OTUs that were assigned to the same species were combined so that sequence reads were summed for each species within each sample and over the entire sample. For certain taxonomic groups, species‐level classifications can be challenging due to shared polymorphisms between closely related species within the barcode region (Schindel and Miller 2005; Purty and Chatterjee 2016; Antil et al. 2022). To address potential misidentification, we checked for correlations between closely related species and corrected the read allocations when necessary. This was done for species from the *Felidae* family, *Bovidae* family (including genera such as *Hippotragus*, *Tragelaphus*, and *Raphicerus*), as well as for various rodent species.

OTU sequences classified as human contaminants or read fragments shorter than 70 base pairs, labeled as “None,” were removed from further analysis. Samples with a total read count > 1000, following these exclusions, were retained for downstream analysis. OTU sequences identified as *Canis* spp. were classified as black‐backed jackal reads. To avoid misidentifying jackal scats, samples in which black‐backed jackal reads made up less than 1% of the total reads were excluded. Due to differences in sequencing depth among samples, species reads were transformed into presence‐absence data for subsequent analysis (Thuo et al. 2019). A prey species was considered present if its read count was 10 or higher, based on the observation that blanks rarely contained more than 10 reads of any species other than *Homo sapiens* , *Canis* spp., or “None.”

### 2.5 Sequencing Summary

Out of the initial 420 fecal samples collected, we retained 63.33% (266 samples) for further analysis following quality control and filtering steps. Of the 154 samples (36.66%) excluded, 12 were lost after merging the forward and reverse reads of each paired‐end sequence, and 27 were removed due to low total read counts (fewer than 1000 reads). Additionally, 17 samples were discarded because black‐backed jackal reads made up less than 1% of the total reads, after removing human and smaller fragmented reads. A further 80 samples were excluded because they were misidentified scats, where reads from other carnivores—such as black‐footed cat ( *Felis nigripes* ), Cape fox ( *Vulpes chama* ), caracal ( *Caracal caracal* ), or leopard ( *Panthera pardus* )—were much higher than those from jackals. Finally, 25 samples were excluded because, although black‐backed jackal reads were predominant, the total read counts for prey species were fewer than 10, leaving no useful information on the jackal diet in those samples, although we cannot completely exclude the possibility of intraspecific predation or scavenging.

In total, the sequencing effort yielded a total of 23,188,331 reads across all retained samples, with an average of 79,959.76 reads per fecal sample. Of these, an average of 49,127.70 reads per sample were identified as originating from *Canis*, accounting for 65.12% of the total reads per sample.

### 2.6 Overview of Scat Sample Processing and Prey Identification

A total number of 266 scat samples were processed for all three sites: Livestock farm (*n* = 62), Game farm (*n* = 99), and Samara Karoo Reserve (*n* = 107).

From the DNA metabarcoding analysis, we were able to taxonomically assign OTUs up to species level for 65 species, and three up to family level (Soricidae, Estrilidae, and Macrosphenidae). The prey species found in the jackal scats varied widely, including large ungulates (> 100 kg, e.g., eland, buffalo ( *Syncerus caffer* ), greater kudu), medium ungulates (26–100 kg, e.g., blesbok, springbok, warthog), and small ungulates (5–25 kg, e.g., steenbok, common duiker), as well as smaller mammals such as mice, rats, shrews, hares, and primates like chacma baboon ( *Papio ursinus* ) and vervet monkey ( *Chlorocebus pygerythrus* ). On rare occasions, we also detected prey taxa such as tortoises, skinks, birds, frogs, hyraxes, and bats. Two large carnivore species, lion and leopard, were identified from the scats, along with eight mesocarnivores: caracal, serval ( *Leptailurus serval* ), Cape fox, small‐spotted genet ( *Genetta genetta* ), black‐footed cat, meerkat ( *Suricata suricatta* ), slender mongoose ( *Galerella sanguinea* ), and bat‐eared fox ( *Otocyon megalotis* ).

### 2.7 Diet Analysis of Black‐Backed Jackals Across the Three Different Land‐Use Sites

To measure and compare the overall dietary composition of black‐backed jackals across the three different sites in the Samara area, we converted the sequence data into two summary metrics: frequency of occurrence (FO) and relative frequency of occurrence (RFO) (Deagle et al. 2018; Thuo et al. 2019; Lu et al. 2021; Shao et al. 2021; Wang et al. 2022). FO was calculated as the number of scat samples in which a given prey taxon was detected. RFO was then calculated as the FO of each prey taxon divided by the total number of prey occurrences across all scat samples, providing a proportional representation of each taxon in the overall diet.

We grouped ungulate species into group sizes based on the adult female body mass of different species (as in Cumming and Cumming 2003), and group sizes included: small (5–25 kg), medium (26–100 kg), and large (> 100 kg) following Hayward et al. (2006) and Mbizah et al. (2012). The rest of the prey species were grouped into taxonomic groups such as rodent, hyrax, bat, reptile, bird, and non‐human primate that included chacma baboons and vervet monkeys. We also added two extra group categories that included “large carnivores” such as lion, leopard, and cheetah, and “other mesocarnivores” such as caracal, serval, Cape fox, small‐spotted genet, and black‐footed cat, because the presence of their DNA inside the scat might be an indication of a scavenging event. In addition, it has been observed that jackals also consume some mesocarnivores, and the latter group of species was therefore also considered as a potential prey group (Bagniewska and Kamler 2013; Bothma 1971b; Klare et al. 2010).

### 2.8 Diversity—And Niche Breadth Indices

To measure the overall species diversity in the diet of jackals across the three sites, we estimated Hill numbers using the iNEXT R‐package (Hsieh et al. 2016), which includes the three most widely used species diversity measures as special cases: species richness (*q* = 0), Shannon diversity (*q* = 1), and Simpson diversity (*q* = 2) (Hsieh et al. 2016). To assess whether sampling captured the entire diversity of the prey community and estimate the diversity under increased sampling effort, we constructed sample‐based accumulation curves. These curves plot the cumulative diversity (for *q* = 0, 1, 2) as a function of the number of samples collected. We used interpolation to estimate the diversity captured by the observed samples and extrapolation to predict diversity at higher levels of sampling effort. Extrapolation extended to double the observed sampling effort to provide robust estimates while avoiding overprediction. Sample‐size and coverage‐size interpolation/extrapolation were performed using the ‘iNEXT’ function, setting to 100 the number of bootstrap replicates for confidence interval estimation.

Furthermore, we estimated the diet diversity of the jackals across the three sites using standardized Levin's index of niche breadth (Fedriani et al. 2000). The formula of diversity of Levin's is *B* = 1/(Σpi^2^), where pi is the proportion of each prey species in the total diet. The standardized formula is: *B*s = (*B*−1)/(*n*−1); where *n* is the maximum number of prey species identified and/or prey groups identified. 𝐵𝑠 ranges from 0 to 1, with lower values suggesting a more specialist diet and higher values suggesting a more generalist diet (Mbizah et al. 2012; Briers‐Louw and Leslie 2020).

### 2.9 Comparison of Species Contribution Across the Land‐Use Sites and Seasons

To identify which specific species or prey groups contributed most to differences in diet composition among the three sites, we applied SIMPER (Similarity Percentage Analysis) from the vegan package (Oksanen et al. 2018). SIMPER decomposes the overall dissimilarity between groups by quantifying the contribution of each species or prey group to the observed differences. This analysis was conducted using (i) the RFO of all detected species, and (ii) the RFO of prey groups.

To compare overall prey consumption across sites and seasons, we used the prey group classifications (e.g., taxonomic or size‐based groups) and fitted a generalized linear mixed‐effects model using the glmmTMB package (Brooks et al. 2017). The total prey count per sample was used as the response variable, with site, season, and prey group included as fixed effects, including all interactions. To account for differences in total prey items among samples and allow valid comparisons of proportional prey group consumption across sites and seasons, we included an offset in the model (Bolker et al. 2009; Dellinger et al. 2011; Fieberg 2024). A negative binomial distribution was specified to accommodate overdispersion in the count data (Hilbe 2011; Zuur et al. 2009). Model fit was evaluated using simulation‐based residual diagnostics from the DHARMa package (Hartig 2024). All statistical analyses were conducted in R (R Core Team 2023).

### 2.10 Prey Group Co‐Occurrence Patterns Within Scat Samples

To investigate whether scavenging on large preys reduced intake on other prey groups, we assessed the likelihood of prey group co‐occurrence in black‐backed jackal diets. We conducted a binomial generalized linear model (GLM) using presence/absence data from individual scat samples. The response variable represented the presence (1) or absence (0) of large ungulate species (> 100 kg) in each scat sample, allowing us to test whether the detection of large ungulates was associated with the co‐occurrence of other prey groups in the same sample. Predictor variables included the presence of other prey groups: rodents, birds, primates, small ungulates, and medium ungulates. Because site and seasonal effects were assessed in earlier analyses, they were excluded here to isolate patterns of co‐occurrence at the individual sample level. The model was checked for overdispersion using Pearson residuals and assessed for overall fit with a Pearson chi‐square test.

## 3 Results

### 3.1 Prey Diversity Across the Three Land‐Use Sites

**FIGURE 3:**
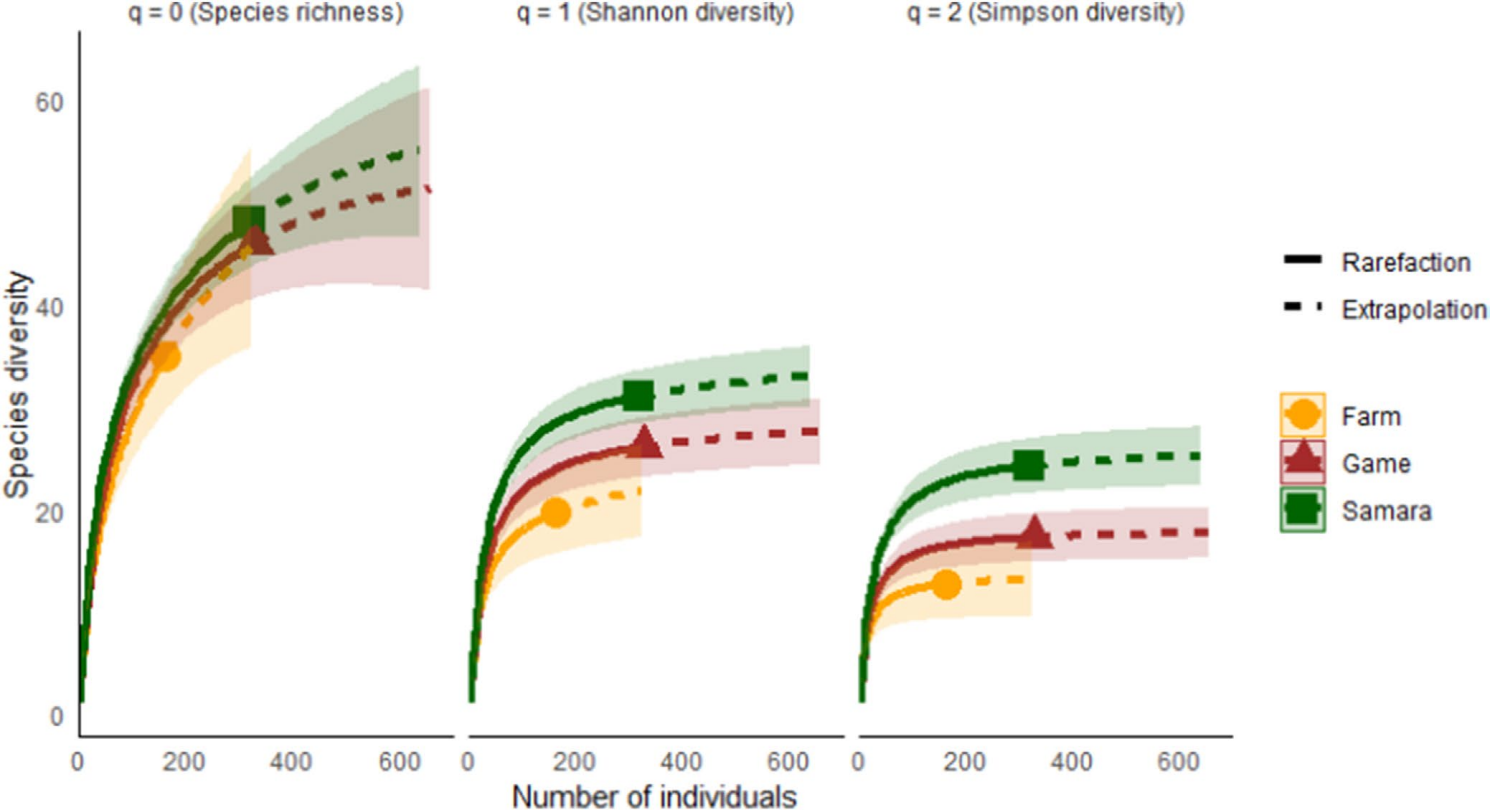
Estimated Hill numbers using the iNEXT package, illustrating three species diversity measures: Species richness (*q* = 0), Shannon diversity (*q* = 1), and Simpson diversity (*q* = 2). Each plot displays coverage‐based interpolation and extrapolation curves for the three diversity measures across the sites.

The diet of the black‐backed jackal showed considerable variation among the sites, as indicated by the range in prey species richness (35–47 species) and Levin's standardized niche breadths (0.17–0.35) (Table 1). While species richness can be influenced by sample size, potentially leading to underestimated dietary diversity, sample‐based rarefaction curves indicated that prey species diversity (Shannon's index) reached an asymptote at the Samara Karoo Reserve and the game farm, with an estimated sample coverage of 90%–95%. However, the livestock farm has not yet reached an asymptote, with only 85% sample coverage, suggesting larger sample sizes are needed to fully capture the black‐backed jackal's dietary diversity. By using rarefaction curves, we adjusted for differences in sample completeness to enable a more accurate comparison of diversity across the sites (Figure 3). The Samara Karoo Reserve exhibited a more diverse and even prey community in jackal diets compared to the game and livestock farms, which showed lower Shannon and Simpson diversities, indicating higher dominance by certain species.

**TABLE 1:** List of prey species detected in 266 scat samples of black‐backed jackals across all three study sites.

Group category	Species (Scientific name)	Samara	Game	Livestock
Larger predator	Lion ( *Panthera leo* )	5 (1.6%)	0	0
Larger predator	Leopard ( *Panthera pardus* )	1 (0.3%)	0	1 (0.6%)
Bat	Large eared free‐tailed bat ( *Otomops martiensseni* )	1 (0.3%)	0	0
Bat	Namibian long‐eared bat ( *Laephotis namibensis* )	1 (0.3%)	0	0
Bird	Helmeted guineafowl ( *Numida meleagris* )	4 (1.3%)	4 (1.2%)	0
Bird	Wabler (Macrosphenidae)	2 (0.6%)	0	0
Bird	Red faced mousebird ( *Urocolius indicus* )	1 (0.3%)	0	0
Bird	Cape crow ( *Corvus capensis* )	1 (0.3%)	2 (0.6%)	0
Bird	Common house martin ( *Delichon urbicum* )	1 (0.3%)	2 (0.6%)	0
Bird	Mannikin (Estrilidae)	1 (0.3%)	0	0
Bird	Chicken (*Gallus gallus domesticus*)	0	0	3 (1.9%)
Bird	Ostrich ( *Struthio camelus* )	0	0	2 (1.3%)
Bird	Jackal buzzard ( *Buteo rufofuscus* )	0	0	1 (0.6%)
Large	Greater kudu ( *Tragelaphus strepsiceros* )	28 (8.9%)	49 (15.1%)	4 (2.5%)
Large	Eland (*Tragelaphus oryx*)	18 (5.7%)	0	0
Large	Roan antelope ( *Hippotragus equinus* )	0	6 (1.9%)	0
Large	Gemsbok ( *Oryx gazella* )	15 (4.8%)	5 (1.5%)	0
Large	Buffalo ( *Syncerus caffer* )	7 (2.2%)	0	0
Large	Burchell's zebra ( *Equus quagga burchellii* )	7 (2.2%)	3 (0.9%)	0
Large	Black wildebeest ( *Connochaetes gnou* )	4 (1.3%)	4 (1.2%)	1 (0.6%)
Large	Cattle ( *Bos taurus* )	1 (0.3%)	5 (1.5%)	12 (7.5%)
Large	Waterbuck ( *Kobus ellipsiprymnus* )	0	2 (0.6%)	0
Large	White rhinoceros ( *Ceratotherium simum* )	0	1 (0.3%)	0
Medium	Springbok ( *Antidorcas marsupialis* )	17 (5.4%)	7 (2.2%)	4 (2.5%)
Medium	Warthog ( *Phacochoerus africanus* )	16 (5.1%)	20 (6.2%)	7 (4.4%)
Medium	pig ( *Sus scrofa domesticus* )	1 (0.3%)	0	3 (1.9%)
Medium	Goat (*Capra aegagrus hircus*)	4 (1.3%)	3 (0.9%)	1 (0.6%)
Medium	Blesbok ( *Damaliscus pygargus phillipsi* )	4 (1.3%)	1 (0.3%)	0
Medium	Sheep ( *Ovis aries* )	0	5 (1.5%)	32 (20%)
Medium	Bushpig ( *Potamochoerus larvatus* )	0	0	1 (0.6%)
Medium	Mountain reedbuck ( *Redunca fulvorufula* )	0	2 (0.6%)	0
Mesocarnivore	Small‐spotted genet ( *Genetta genetta* )	4 (1.3%)	6 (1.9%)	3 (1.9%)
Mesocarnivore	Cape fox ( *Vulpes chama* )	3 (1.0%)	1 (0.3%)	5 (3.1%)
Mesocarnivore	Bat‐eared fox ( *Otocyon megalotis* )	0	1 (0.3%)	1 (0.6%)
Mesocarnivore	Black‐footed cat ( *Felis nigripes* )	0	0	1 (0.6%)
Mesocarnivore	Meerkat ( *Suricata suricatta* )	0	0	1 (0.6%)
Mesocarnivore	Serval ( *Leptailurus serval* )	0	3 (0.9%)	0
Mesocarnivore	Caracal ( *Caracal caracal* )	0	4 (1.2%)	0
Mesocarnivore	Slender mongoose ( *Galerella sanguinea* )	0	1 (0.3%)	0
Primate	Vervet monkey ( *Chlorocebus pygerythrus* )	16 (5.1%)	7 (2.2%)	1 (0.6%)
Primate	Chacma baboon ( *Papio ursinus* )	10 (3.2%)	12 (3.7%)	0
Reptile	Cape river frog (*Amieta fuscigula*)	2 (0.6%)	1 (0.3%)	0
Reptile	Bubbling kassina ( *Kassina senegalensis* )	0	1 (0.3%)	0
Reptile	Cape legless skink (*Acontias meleagris*)	1 (0.3%)	0	0
Reptile	Leopard tortoise (*Stigmachelys pardalis*)	1 (0.3%)	2 (0.6%)	1 (0.6%)
Rodent	Single‐striped grass mouse ( *Lemniscomys rosalia* )	22 (7.0%)	32 (9.9%)	14 (8.8%)
Rodent	Southern multimammate mouse ( *Mastomys coucha* )	15 (4.8%)	11 (3.4%)	0
Rodent	Cape hare ( *Lepus capensis* )	14 (4.4%)	17 (5.2%)	10 (6.3%)
Rodent	Natal red rock hare ( *Pronolagus crassicaudatus* )	0	2 (0.6%)	2 (1.3%)
Rodent	Karoo bush rat ( *Myotomys unisulcatus* )	10 (3.2%)	17 (5.2%)	2 (1.3%)
Rodent	Southern african pygmy mouse ( *Mus minutoides* )	9 (2.9%)	11 (3.4%)	3 (1.9%)
Rodent	Namaqua rock rat ( *Micaelamys namaquensis* )	8 (2.5%)	17 (5.2%)	6 (3.8%)
Rodent	Cape short‐eared gerbil ( *Desmodillus auricularis* )	5 (1.6%)	0	0
Rodent	Desert pygmy mouse ( *Mus indutus* )	5 (1.6%)	2 (0.6%)	1 (0.6%)
Rodent	South African springhare ( *Pedetes capensis* )	3 (1.0%)	0	2 (1.3%)
Rodent	Common mole‐rat ( *Cryptomys hottentotus* )	2 (0.6%)	4 (1.2%)	1 (0.6%)
Rodent	Laminate vlei rat ( *Otomys laminatus* )	2 (0.6%)	21 (6.5%)	15 (9.4%)
Rodent	Hairy footed gerbil ( *Gerbillurus paeba* )	2 (0.6%)	1 (0.3%)	0
Rodent	Shrew family (Soricidae)	2 (0.6%)	3 (0.9%)	1 (0.6%)
Rodent	Southern african vlei rat ( *Otomys irroratus* )	0	7 (2.2%)	3 (1.9%)
Rodent	Ground squirrel ( *Xerus inauris* )	1 (0.3%)	0	0
Rodent	House mouse ( *Mus musculus* )	1 (0.3%)	0	0
Rodent	Round‐eared elephant shrew ( *Macroscelides proboscideus* )	0	0	1 (0.6%)
Rodent	Western rock elephant shrew ( *Elephantulus rupestris* )	0	1 (0.3%)	0
Small	Common duiker ( *Sylvicapra grimmia* )	16 (5.1%)	7 (2.2%)	4 (2.5%)
Small	Cape grysbok ( *Raphicerus melanotis* )	15 (4.8%)	5 (1.5%)	0
Small	Steenbok ( *Raphicerus campestris* )	6 (1.9%)	5 (1.5%)	11 (6.9%)
Hyrax	Cape rock hyrax ( *Procavia capensis* )	0	1 (0.3%)	0
	Total individuals	315	324	161
	Total samples	107	99	62
	Levin's standardized niche breadths per species	0.35	0.25	0.17
	Levin's standardized niche breadths per prey group category	0.38	0.25	0.29

*Note:* The table includes the total count of each species detected, with the relative frequency of occurrence (RFO) presented as a percentage in brackets. Each species is also categorized into a broader prey group used for dietary analysis.

**FIGURE 4:**
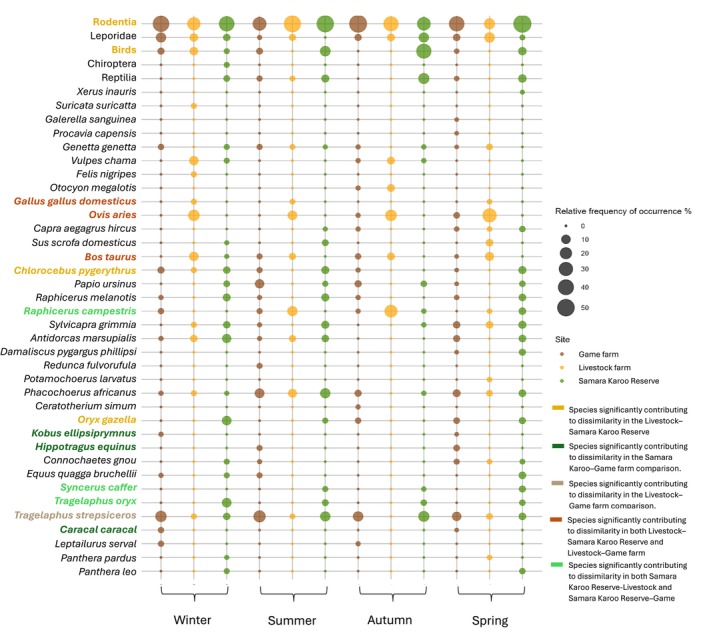
Bubble plot representing the relative frequency of occurrence (%) of each species in the diet of black‐backed jackals across four collection periods at three different sites. Colored species names denote significant associations with specific sites, with colors corresponding to those contributing to dissimilarity in pairwise site comparisons.

The bubble plot representing the species across the three sites (Figure 4) further illustrates the species richness overlaps: in addition to the 21 shared species, the Samara Karoo Reserve and the game farm shared 10 additional species, while the livestock farm had an additional three species in common with both the Samara Karoo Reserve and the game farm. Among the species shared across the sites, half of the rodent species—which were the most frequently detected—were found at all locations. In addition, ungulates such as warthog and springbok were common to all sites, along with common duiker, steenbok, and greater kudu.

### 3.2 The Overall Contribution of Different Prey Species Across the Land‐Use Sites and Seasons

The SIMPER analysis identified key species contributing to dissimilarities between sites (Table S2). Between the game farm and livestock farm, sheep (*p* = 0.009), cattle (*p* = 0.030), and chicken (*p* = 0.021) were significant contributors. Comparisons between the livestock farm and Samara Karoo Reserve highlighted livestock species such as sheep (*p* = 0.001), cattle (*p* = 0.001), and chicken (*p* = 0.014), along with eland (*p* = 0.002). Fewer species contributed to the dissimilarity between the game farm and Samara Karoo Reserve, with eland (*p* = 0.001) and buffalo (*p* = 0.012) being the strongest contributors. Prey size group analysis mirrored these patterns, with medium and large ungulates driving differences between the livestock and game farms, and large ungulates and primates between the livestock farm and Samara Karoo Reserve. No significant differences were observed between the game farm and Samara Karoo Reserve, although rodents showed a near‐significant trend (*p* = 0.071).

The generalized linear mixed‐effects model, which accounted for both site and season, confirmed that prey size group consumption varied significantly, with interactions between site, season, and prey group. Medium‐sized ungulates were higher on the livestock farm during winter (game farm—livestock farm, *p* = 0.014) and spring (game farm—livestock farm, *p* = 0.008; Samara Karoo Reserve—livestock farm, *p* = 0.024). Larger ungulates were higher on the game farm compared to Samara Karoo Reserve in summer (*p* = 0.036), while rodents were higher in Samara Karoo Reserve compared to the game farm in winter (*p* = 0.004). Smaller ungulates were higher on the livestock farm in autumn compared to the game farm (*p* = 0.047).

**FIGURE 5:**
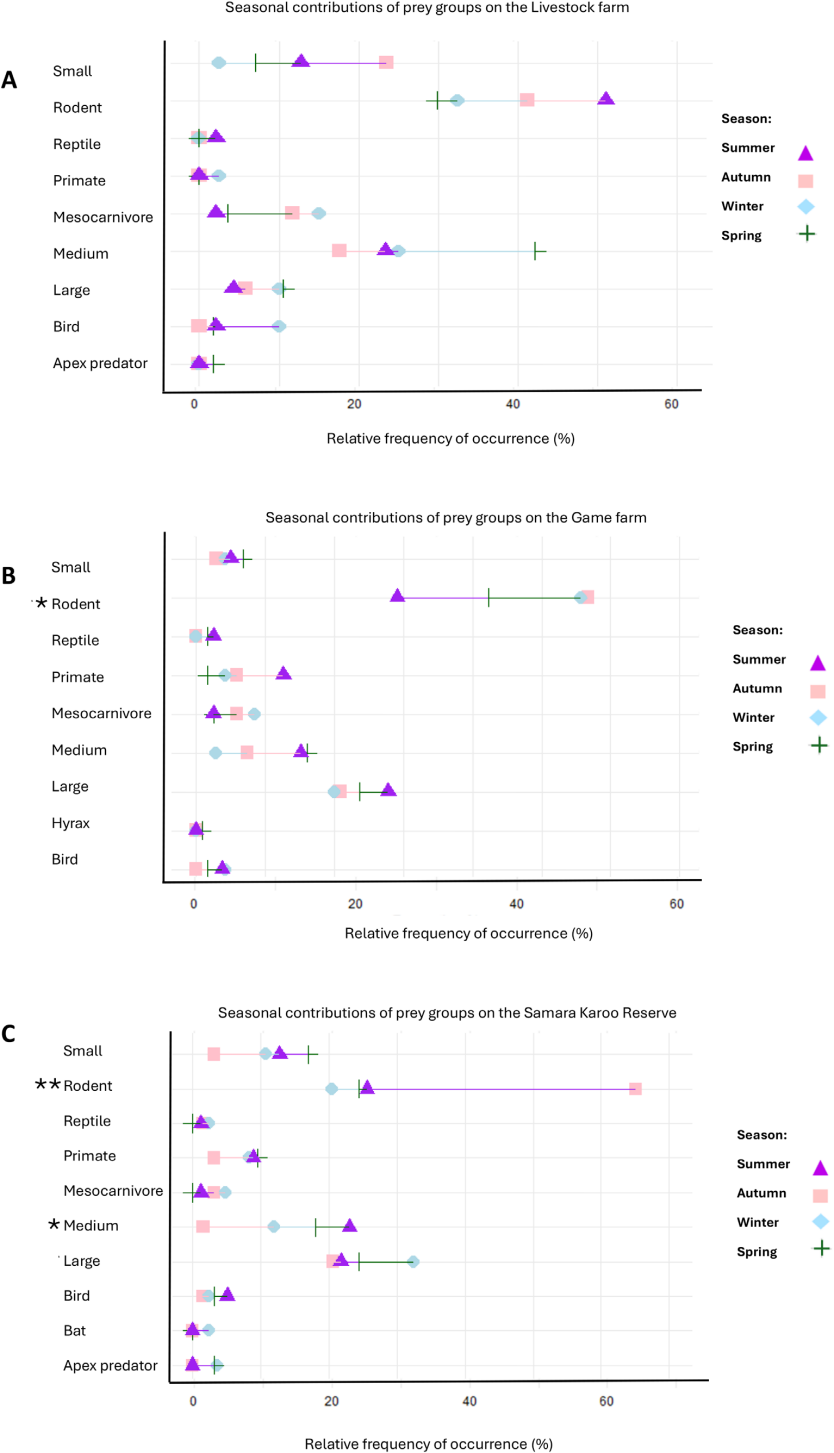
Dot plots illustrating the variation in the proportion of each prey group—including the apex predator group—across the monthly collection periods at (A) the livestock farm, (B) the game farm, and (C) the Samara Karoo Reserve. Lines connecting the month symbols indicate changes in percentages between collection periods. Asterisks (*p < 0.05, **p < 0.001) mark prey groups with significant differences between the seasons as determined by the generalized linear mixed‐effects model.

Seasonal patterns within sites showed that the game farm had the highest rodent consumption in autumn compared to summer (*p* = 0.048; Figure 5B), while Samara Karoo Reserve peaked in autumn relative to all other seasons (autumn–summer, *p* = 0.004; autumn–spring, *p* = 0.002; autumn–winter, *p* = 0.0009; Figure 5C). Medium‐sized ungulates at Samara Karoo Reserve were consumed more in summer than in autumn (*p* = 0.045; Figure 5C). The livestock farm exhibited no significant seasonal variation in prey group proportions (Figure 5A).

Residual diagnostics indicated the negative binomial GLMM with an offset term was appropriate: dispersion was slightly below 1 (0.887, *p* = 0.074), zero‐inflation was not significant (ratioObsSim = 0.988, *p* = 0.274), and residuals followed the expected distribution (*D* = 0.018, *p* = 0.495).

### 3.3 Prey Group Co‐Occurrence Patterns Within Scat Samples

A binomial regression model was fit to assess the factors influencing the detection of large ungulates in black‐backed jackal scats. The model included the following predictors: medium ungulate, rodent, small ungulate, bird, and primate. Reptiles and bats were excluded from the model due to their low occurrence in the scats, as they were detected in only a very small proportion of the samples, with most samples showing no presence of these prey groups. The binomial GLM indicated that when all other prey groups were absent, larger ungulates were significantly more likely to be present (1.11 ± 0.27, *z* = 4.13, *p* < 0.0001), corresponding to a 75% likelihood of detection. The presence of medium ungulates significantly decreased the probability of large ungulate detection (−0.88 ± 0.29, *z* = −3.03, *p* < 0.01), reducing the odds of occurrence by approximately 59%. Similarly, the presence of rodents (−1.16 ± 0.28, *z* = −4.18, *p* < 0.0001) reduced the odds of detecting large ungulates by approximately 69%, while the presence of primates (−0.98 ± 0.38, *z* = −2.57, *p* = 0.01) reduced the odds by around 62%. Small ungulates had no statistically significant effect on large ungulate presence (0.40 ± 0.34, *z* = 1.17, *p* = 0.24), although their presence was associated with a ~49% increase in odds. Birds likewise had no significant effect (−0.98 ± 0.38, *z* = 0.64, *p* = 0.53), with a non‐significant 62% decrease in the odds of large ungulate detection.

Model fit was good, with no evidence of overdispersion (dispersion = 1.02). A likelihood ratio test comparing the full model to the null model showed a significant improvement in model fit (*χ* ^2^ = 28.20, *p* < 0.0001), suggesting that the predictors significantly contribute to the model's explanatory power. The Pearson chi‐square test indicated no lack of fit (*p* = 0.386).

## 4 Discussion

Our findings highlight the behavioral flexibility of jackals, demonstrating their ability to switch between actively hunting ungulates, scavenging on carcasses left by larger carnivores, and targeting small mammals. Interestingly, our study on the dietary response of black‐backed jackals is among the few conducted at the landscape level, considering how prey presence can vary across neighboring land uses (but see Klare et al. 2010; Kamler, Klare, and Macdonald 2012; Forbes 2011; Jansen 2016; and Drouilly et al. 2018). Although species richness was broadly similar across sites, the composition of prey detected in jackal scats differed notably. These differences can be attributed to several ecological and behavioral factors, including prey vulnerability, energy demands, seasonal reproductive cycles, and interspecific interactions (Atkinson et al. 2002; Loveridge and Macdonald 2003; Van der Merwe et al. 2009). Exploring these factors in detail allows us to better understand how jackals adapt their foraging strategies across dynamic landscapes.

### 4.1 Seasonal Shifts and Opportunistic Foraging Across Contrasting Land Uses

Jackal diets ranged from broad and relatively even at the nature reserve, where a wide variety of species contributed to the diet, to more specialized diets at the game and livestock farms, where particular prey species dominated (Figure 3).

On the livestock farm, medium‐sized ungulates, particularly sheep, were the most consumed prey (Figure 4). As sheep are the most prevalent species on the livestock farm and do not exhibit effective anti‐predator behaviors (Kruuk 1972), they are highly vulnerable to jackal depredation. Similar patterns have been reported in the Free State, Namaqualand, and Central Karoo, where sheep frequently dominate jackal diets (Kamler, Klare, and Macdonald 2012; Jansen 2016; Drouilly et al. 2018).

Our study showed that sheep consumption on the livestock farm peaked in spring (Figure 4), when lambs, being smaller and more vulnerable, are expected to be easier prey for jackals than adult sheep. Although lambs were present year‐round due to farm management practices (with rams left in the herds), spring likely remains a peak lambing period in the Karoo as veld conditions are more favorable at this time. This coincides with the period when female jackals are suckling pups, and the higher levels of sheep consumption during spring are therefore likely driven by both the increased availability of lambs and the elevated energetic demands of pup‐rearing, especially for lactating females (Bingham and Purchase 2002; Green et al. 2022). Interestingly, elevated consumption of medium‐sized prey was also recorded in winter on the livestock farm, a pattern not evident at the game farm or Samara Karoo Reserve (Figure 5), likely reflecting the continuous availability of lambs.

In contrast, during autumn and summer, sheep contributed less to jackal diets, while steenbok consumption peaked on the livestock farm (Figure 4). This aligns with steenbok reproductive cycles, where their 5.5‐month gestation results in births concentrated in autumn and summer (Kingdon 1997; Kamler, Klare, and Macdonald 2012). Kamler, Klare, and Macdonald (2012) similarly reported peak steenbok consumption in autumn. Steenbok have the second widest distribution of all African antelope, largely due to their ability to persist in areas where larger antelopes have declined as a result of human pressures (Furstenburg 2005). This is exemplified on the livestock farm, where high stocking rates of sheep, cattle, and Angora goats have altered the landscape. Their capacity to exploit small patches of natural habitat (Furstenburg 2005; Jansen van Vuuren et al. 2022) likely increases their encounter rate with jackals in these modified environments.

Other livestock species such as pigs and chickens were also detected in scats (Figure 4) from the livestock farm, probably linked to farm personnel living on‐site. Cattle were also detected, consistent with findings from KwaZulu‐Natal, where jackals preyed on newborn calves and occasionally on weakened adults (Humphries et al. 2016). These detections highlight that jackals may exploit a wide range of livestock when opportunities arise.

Following observations of high predation on sheep lambs and steenbok, jackals appear to exploit opportunities presented by smaller, young, or otherwise easily captured ungulates. Multiple studies have shown that jackals specialize in hunting newborn ungulates, particularly those classified as ‘hiders’ that conceal their young in tall vegetation during the first few weeks after birth (Klare et al. 2010). This behavior appears consistent across various land‐use types. Most ungulate species tend to give birth when food resources are more abundant and there is sufficient cover for their young, conditions that often align with the rainy seasons of spring and summer (Ogutu et al. 2014). Jackals not only actively hunt for newborn ungulates but are also known to scavenge carcasses left by larger carnivores (Kamler et al. 2010; Brassine and Parker 2012; Yarnell et al. 2013; Hayward et al. 2017).

On the game farm, there is a noticeable increase in the consumption of medium‐sized ungulates, such as blesbok, springbok, and warthog, during spring and summer (Figure 4). This period also marks a peak in the consumption of common duiker, a smaller‐sized ungulate (Figure 4). The consumption of large ungulates remains relatively stable throughout the year, with subtle trade‐offs observed among hider species such as greater kudu, roan antelope, and gemsbok (Figures 4 and 5). This stability likely reflects the year‐round presence of greater kudu. The absence of larger carnivores on the game farm likely allows jackals to focus on kudu fawns, as adult greater kudu is too large for jackals to prey upon. The peak in greater kudu births typically coincides with the wet season, explaining their increased consumption during the summer months (Owen‐Smith 1997). Additionally, fieldwork observations recorded adult greater kudu carcasses on the game farm, likely resulting from natural causes such as hypothermia and pneumonia (Furstenberg 2022). These carcasses may provide an alternative food source for jackals when fawns are less available, contributing to another peak in greater kudu consumption during the winter months (Figure 4).

In the third site, at the Samara Karoo Reserve, large ungulate consumption was higher during the winter months (Figure 5). Species like eland and gemsbok contribute more to the jackal diet during this period (Figure 4). Since winter is not a favorable season for giving birth, this suggests that jackals at Samara Karoo Reserve may access these larger ungulates primarily through scavenging, likely feeding on carcasses left by lions. However, similar to the game farm and livestock farm, medium‐sized ungulate consumption peaks during spring and summer (Figure 5), with notable increases in blesbok and warthog consumption (Figure 4). These findings reinforce the idea that jackals use a flexible feeding strategy, preying on newborn ungulates during spring and summer while capitalizing on scavenging opportunities during winter. This adaptability allows them to exploit seasonal shifts in prey availability, aligning with previous research on their preference for ungulates that conceal their young (Klare et al. 2010; Hayward et al. 2017).

It has to be highlighted that rodents were the most frequently consumed prey across all sites, but seasonal patterns varied. At the game farm, rodent consumption peaked between autumn and winter and declined in spring and summer as the larger wild ungulates became more prominent in the diet (Figure 5). Similarly, at Samara Karoo Reserve, rodent consumption peaked in autumn, but decreased in winter, and began to slowly rise again in spring and summer (Figure 5). In contrast, the livestock farm showed no statistically significant seasonal variation in rodent consumption (Figure 5). However, the data indicated a decline in rodent consumption during spring, coinciding with an increase in sheep consumption (Figure 5). This lack of statistical significance may partly reflect reduced sample sizes in autumn and winter, as several samples were excluded during DNA filtering after being identified as originating from other mesocarnivores, including the small‐spotted genet, Cape fox, and black‐footed cat.

The daily activity patterns of rodents, which align with those of black‐backed jackals, may explain their prevalence in jackal diets (Rowe‐Rowe 1983; Ferguson et al. 1988). Rainfall patterns in the Karoo region, where rainfall peaks in autumn and spring, may result in a time lag for vegetation growth, offering a late autumn and early winter food flush for rodents (Feng et al. 2023). A notable aspect of this study is the increased presence of primarily nocturnal rodent species during the autumn–winter period, which coincides with heightened activity levels in black‐backed jackals, as evidenced by the findings of Kamler et al. (2019) and Ferguson et al. (1988). This time frame corresponds with the jackals' mating season, characterized by intensified movement and territorial patrols (Ferguson et al. 1988; Kamler et al. 2019). The prolonged activity cycles during the dry season (May–August) likely enhance the chances of encountering rodents. Conversely, during the wet season, jackals may redirect their focus to energy‐rich resources, such as ungulate fawns, particularly during the pup‐rearing season (Moehlman 1987).

Another notable difference in jackal diet was the presence of primates, particularly at the Samara Karoo Reserve (Figures 4 and 5). Vervet monkeys occurred in 14.15% of scats and chacma baboons in 9.43%, whereas on the game farm, chacma baboons (12%) were detected more frequently than vervet monkeys (7%). Six scats containing vervet monkey DNA also contained baboon DNA, suggesting possible scavenging or indirect predation. Previous work at Samara Karoo Reserve using hair analysis also identified vervet monkeys as frequent prey in jackal diet (Van de Ven et al. 2013). Historically, black‐backed jackals have been considered potential predators of vervet monkeys (Cheney and Seyfarth 1981; Struhsaker 1967). Chacma baboons, as flexible omnivores, are known to kill and consume vervet monkeys, indicating potential competition along the seasonal Melk River (Figure 2). In contrast, this primate association was absent on the game farm, possibly due to the availability of more streams and valleys, which may allow the two species to occupy separate areas (Figure 2). Given this interaction, it is plausible that the jackals could have scavenged from vervet monkey carcasses killed and partially consumed by baboons in the Samara Karoo Reserve. On the livestock farms, both primates were absent from jackal scats, likely due to their status as pests, with farmers actively deterring or killing them.

### 4.2 Influence of Large Carnivores and Mesocarnivores in Jackal Diets

Despite the presence of large carnivores at the Samara Karoo Reserve, their influence on jackal diet composition appeared minimal. Large ungulates (> 100 kg) were detected in similar proportions in jackal scats from both Samara (25.40%) and the carnivore‐free game farm (23.22%) (Figure 5). This suggests that the mere presence of apex predators does not necessarily increase scavenging opportunities or substantially shift jackal dietary preferences. Nevertheless, scavenging opportunities likely arise on reserves like Samara, particularly from lion kills of large‐bodied prey such as buffalo and eland—species that are not only inaccessible to jackals as adults, but even their calves are likely too large and well‐defended to be viable prey (Hayward and Kerley 2005; Annear et al. 2023). Accessing such carcasses allows jackals to exploit high‐energy resources without incurring the substantial costs associated with hunting. This may reduce the need to pursue more energetically demanding prey such as medium‐sized ungulates or newborn fawns (Klare et al. 2010).

The role of larger carnivores in shaping jackal diets remains contested in the literature. Some studies have found significant dietary shifts associated with larger carnivore presence (Fourie et al. 2015), while others report little to no effect (Brassine and Parker 2012; Yarnell et al. 2013). Such variation likely reflects differences in predator densities, scavenging opportunities, and prey availability across landscapes (Ramnanan et al. 2016; Minnie, Zalewski, et al. 2018). In our study, large carnivore density at the Samara Karoo Reserve was relatively low, including only one lion pride, a cheetah coalition, a female cheetah with sub‐adults, and potentially a roaming leopard pair—without any spotted hyena ( *Crocuta crocuta* ) or brown hyena ( *Parahyaena brunnea* ). In contrast, in another study at the Welgevonden Private Game Reserve, where large carnivore density is higher, jackals relied more consistently on large ungulates year‐round (44% contribution to diet; Roberts et al. 2025, unpublished). At that site, large ungulate DNA in jackal scats also frequently co‐occurred with DNA from other carnivores, particularly during the dry season, suggesting increased scavenging opportunities during resource‐scarce periods.

Another notable observation is the detection of other mesocarnivores in jackal scats, particularly on the livestock farm (Figures 4 and 5), highlighting complex trophic dynamics. Black‐backed jackals are known to consume or kill at least 17 species of smaller carnivores from six families, including the bat‐eared fox, Cape fox, caracal, black‐footed cat, small‐spotted genet, and yellow mongoose ( *Cynictis penicillata* ) (Bagniewska and Kamler 2013), and our results support this. Mesocarnivore populations often increase when larger predator numbers decline, a phenomenon known as ‘mesopredator release’ (Crooks and Soulé 1999; Prugh et al. 2009). As a result, on both the livestock and game farms, where larger carnivores are absent, the black‐backed jackal may have assumed the role of the top predator. This shift has led to greater interactions among mesocarnivores, with jackals either scavenging from other mesocarnivores' kills or preying on the mesocarnivores themselves. Kamler, Stenkewitz, et al. (2012) found that jackals suppress populations of Cape foxes and bat‐eared foxes, and that these species in turn adopt spatial and temporal avoidance strategies. Our observations of increased relative occurrence of other mesocarnivores on livestock and game farms may reflect similar trophic dynamics, with jackals interacting more with these species. On livestock farms, persecution of jackals by farmers likely reduces their densities, potentially allowing mesocarnivores such as Cape foxes to increase in abundance.

### 4.3 Prey Size Selection and Optimal Foraging Strategies

Across all sites in our study, jackals appeared to follow an optimal foraging strategy (Pyke et al. 1977; Pyke 1984): targeting small, easily captured prey like rodents when hunting actively, while opportunistically scavenging larger prey when available. Although no strict prey trade‐off was observed, strong negative correlations between large ungulates and smaller prey groups (rodents, medium ungulates, and primates), as revealed by our binomial GLM, suggest that jackals adjust their diet according to prey availability. Notably, the probability of detecting large ungulates was highest (≈75%) when smaller prey groups were absent, but declined significantly when rodents, medium ungulates, or primates were present.

This flexibility highlights that different prey groups complement each other in the diet, rather than substituting for one another. Larger ungulates (> 100 kg) are generally inaccessible for jackals unless they are scavenged from carcasses or newborn fawns, while medium ungulates (< 100 kg) could potentially be subdued by a pair of jackals (Van der Merwe et al. 2009; Klare et al. 2010; Kamler et al. 2010). However, the handling costs of capturing medium ungulates are high, which may be compounded when access to carcasses is limited by larger carnivores. While jackals exploit larger prey when the opportunity arises, smaller prey—particularly rodents—still play a crucial role in their diet. Rodents were the most frequently consumed prey group across all sites, contributing 32%–45% to the diet (Figure 5). Their consistent presence likely reflects both their abundance and ease of capture, despite offering less energy per individual. This aligns with an optimal foraging strategy, where jackals balance energy intake with handling and risk costs.

### 4.4 Strengths and Limitations

We used DNA metabarcoding to assess black‐backed jackal diets across contrasting landscapes, providing good taxonomic resolution and efficient identification of vertebrate prey. By targeting vertebrates, using the degenerate primer 12SV5 (Riaz et al. 2011), we captured the primary dietary components most relevant to understanding differences across sites. This choice inevitably excluded fruits and invertebrates, which, although occasionally detected in scats, generally contribute little to the jackal diets and are consumed opportunistically and seasonally (Hiscocks and Perrin 1988; Do Linh San et al. 2009; Klare et al. 2010; Forbes 2011). Our focus was therefore on the main vertebrate prey and the differences across sites. Some scat samples may therefore have yielded limited vertebrate DNA if insects or plant matter dominated their content. Nonetheless, our focus was on the main vertebrate prey preference across land uses.

DNA metabarcoding offers powerful strengths, particularly fine‐scale prey detection and the ability to process large sample sets efficiently, but it also presents interpretive challenges. Relative read abundance may not directly represent prey biomass due to variation in digestion rates, DNA persistence, and amplification bias (Thuo et al. 2019). While this limits quantitative inference, it does provide robust insight into prey prevalence and composition. Interpreting these results with caution is essential, as a single scat may represent multiple meals, and even small tissue samples (e.g., placenta) can yield strong DNA signals despite representing minimal biomass. Future research should continue to refine these techniques, aiming to better model dietary intake and address the complexity of interpreting read abundance.

A further limitation concerns prey availability data. Exact prey abundance estimates within each site were not available for all species. Aerial counts of large ungulates provided some context for populations on the game farm and Samara Karoo Reserve, but since jackal diets include a wide range of prey, including hares, rodents, steenbok, and common duiker, these counts were insufficient for a comprehensive comparison. Therefore, we focused on the presence and availability of prey species across the different farms. While full abundance surveys, including small mammals, ungulates, and mesocarnivores, would enhance understanding of prey preference, such efforts represent large, resource‐intensive projects beyond the scope of this study. Thus, we focused on comparing prey presence across farms rather than attempting detailed prey preference analyses.

### 4.5 Management Implications

Our data suggest that black‐backed jackals can potentially move across land‐use boundaries, blurring the lines between wild and domestic landscapes. DNA from domestic livestock (sheep, goats, pigs, cattle) was detected on both farms and within the Samara Karoo Reserve. Notably, white rhinoceros DNA was found near the game farm–reserve boundary, indicating that jackals can access resources across different land uses. While our observations suggest that jackals may be crossing boundaries, it is important to consider that this behavior could be opportunistic rather than intentional. Access is not always guaranteed, as fences are typically monitored and maintained. For example, Samara Karoo Reserve employs multiple electrified fences, patrolled daily to identify weak spots and prevent animals from entering or escaping. Though rare, these instances indicate that jackals in protected areas may exploit anthropogenic resources from neighboring farms, likely gaining access through occasional breaches or weak points in the fences.

High reliance on livestock underscores the potential for human–wildlife conflict, particularly during lambing seasons. Jackals' movement between protected areas and farms complicates population control, especially where larger carnivores have been removed. Without top‐down regulation, jackals may persist at higher densities, consistent with sink–source dynamics and mesocarnivore release theory (Ritchie and Johnson 2009; Newsome and Ripple 2015). For example, culled individuals in areas with abundant food are often replaced by conspecifics emigrating from higher‐density areas, and jackals can compensate for increased mortality with higher reproductive rates, explaining their persistence on private farmlands despite intensive human persecution (Minnie, Avenant, et al. 2016; Minnie, Gaylard, and Kerley 2016).

Currently, no active conservation measures target jackals; management generally focuses on population control to reduce predation on valuable ungulates. However, the effectiveness of these strategies remains unclear. Monitoring populations is essential to understand distribution, abundance, and the impacts of predator management, providing a foundation for informed, site‐specific conservation strategies that mitigate human–wildlife conflict. Population density and social structure likely influence dietary patterns, and although not directly measured here, they are recognized as important ecological factors.

## 5 Conclusion

Overall, black‐backed jackals demonstrate remarkable dietary flexibility, integrating a wide range of prey to meet their energetic demands and shifting with prey availability across different landscapes. Large ungulates provide high‐energy rewards when accessible, while small prey offer consistent, low‐risk resources. This balance reflects an optimal foraging strategy, allowing jackals to adapt to diverse ecological contexts and highlighting the importance of dynamic, site‐specific management approaches.

Studying the ecology of black‐backed jackals is like pursuing a moving target: their adaptability, combined with their mobility across varied land‐use types, enables them to exploit a broad prey base, a trait that supports population growth. As human land use reshapes landscapes, these dynamics become even more pronounced. Livestock farms promote domestic animals at the expense of wild prey, game farms prioritize valuable hunting ungulates, and nature reserves sustain more diverse wildlife communities. Reflecting these contrasts, our study revealed that jackals on livestock farms primarily consumed sheep, on game farms relied heavily on greater kudu (likely fawns and weakened individuals), and on the nature reserve exhibited the broadest diet, supplemented by carrion from apex predators. These patterns underscore the ecological resilience of jackals across both natural and human‐modified systems.

## Author Contributions

**Megan Roberts:** conceptualization (lead), data curation (lead), formal analysis (lead), investigation (lead), methodology (equal), project administration (lead), software (lead), validation (lead), visualization (lead), writing – original draft (lead), writing – review and editing (lead). **Fanny Degrugillier:** methodology (equal). **Locadia Dzingwena:** methodology (supporting). **Hervé Fritz:** conceptualization (supporting), formal analysis (supporting), funding acquisition (supporting), investigation (supporting), methodology (supporting), supervision (equal), validation (supporting), visualization (supporting), writing – review and editing (supporting). **Virginie Rougeron:** conceptualization (supporting), formal analysis (supporting), investigation (supporting), methodology (supporting), resources (equal), supervision (equal), validation (supporting), visualization (supporting), writing – review and editing (supporting). **Franck R. Prugnolle:** conceptualization (supporting), data curation (supporting), formal analysis (supporting), funding acquisition (lead), investigation (supporting), methodology (supporting), resources (equal), supervision (lead), validation (supporting), visualization (supporting), writing – review and editing (supporting).

## Conflicts of Interest

The authors declare no conflicts of interest.

## Supporting information


**Data S1:** Supporting Information

## Data Availability

Raw sequence reads have been submitted to the NCBI Sequence Read Archive (SRA) under BioProject ID: SUB14897117. However, they are not yet publicly accessible and are pending upload assistance from the affiliated institution. In the interim, raw read files have also been uploaded to the Dryad Digital Repository. All processed data, including sample metadata, filtered read counts per sample, species identifications, and final presence/absence matrices used in statistical analyses, are available via Dryad DOI: https://doi.org/10.5061/dryad.n02v6wx8g.
