# Correction to “Dietary Response of Black‐Backed Jackals (*Lupulella mesomelas*) to Contrasted Land Use”

**DOI:** 10.1002/ece3.73196

**Published:** 2026-03-01

Roberts, M., F. Degrugillier, L. Dzingwena, H. Fritz, V. Rougeron, and F. R. Prugnolle. 2025. “Dietary Response of Black‐Backed Jackals (*Lupulella mesomelas*) to Contrasted Land Use.” *Ecology and Evolution* 15, no. 10: e72186. https://doi.org/10.1002/ece3.72186.

In the Methods section, under “DNA extraction and PCR amplification” describing amplicon library preparation, the forward 12SV5degForward primer sequence was reported incorrectly. The text currently states: “12SV5degForward: 5′‐YRGACAAGTCCACAATCTAG‐3′.” This should have read as follows: “12SV5degForward: 5′‐YRGAACAGGCTCCTCTAG‐3′.”

The reverse primer sequence is correct as published. The correct primer sequences are also accurately reported in Table S1 in the Supporting Information section. This error is limited to the in‐text description and does not affect the sequencing, data analyses, or conclusions of the study.

We apologize for this error.

