# Supplementary material for: Dietary Response of Black‐Backed Jackals (Lupulella mesomelas) to Contrasted Land Use
Source: Ecol Evol. 2025 Oct 9;15(10):e72186. doi: 10.1002/ece3.72186 (PMC12508620; doi:10.1002/ece3.72186)
Supplement: Supplementary file 1 — Data S1: Supporting Information [file ECE3-15-e72186-s001.docx]

**Supplementary Table 1:** Primers used in this study. The 12SV5deg primer sets are based on the original universal primer for vertebrates by Riaz et al. (2012), modified with degenerate bases to accommodate a broader range of species. The three sets of forward and reverse 12SV5 primers include 8-base-pair inline tags and/or P7/P5 overhangs, enabling index PCRs to incorporate Illumina adapter and index sequences. Tag sequences are represented as N.

| P5-N-12SV5deg-Forward | 5’-GTC TCG TGG GCT CGG AGA TGT GTA TAA GAG ACA G N YRG AAC AGG CTC CTC TAG-  3’ |
| --- | --- |
| P5-NN-12SV5deg-Forward | 5’-GTC TCG TGG GCT CGG AGA TGT GTA TAA GAG ACA G NN YRG AAC AGG CTC CTC  TAG-3’ |
| P5-NNN-12SV5deg-Forward | 5’-GTC TCG TGG GCT CGG AGA TGT GTA TAA GAG ACA G NNN YRG AAC AGG CTC CTC  TAG-3’ |
| P7-N-12SV5deg-Reverse | 5’-TCG TCG GCA GCG TCA GAT GTG TAT AAG AGA CAG N TTA GAT ACC CCA CTA TGY-3’ |
| P7-NN-12SV5deg-Reverse | 5’-TCG TCG GCA GCG TCA GAT GTG TAT AAG AGA CAG NN TTA GAT ACC CCA CTA TGY-  3’ |
| P7-NNN-12SV5deg-Reverse | 5’-TCG TCG GCA GCG TCA GAT GTG TAT AAG AGA CAG NNN TTA GAT ACC CCA CTA TGY-  3’ |

**Supplementary Table 2:** Results of SIMPER identifying the species that contributed most to the differences in dietary composition of black-backed jackals across the three study sites. The table displays the contribution of each species to dissimilarity and associated p-values, indicating the strength of each species' contribution to site-based dietary differences.

| Species | Game | Livestock | | Samara Karoo Reserve |
| --- | --- | --- | --- | --- |
| Bat-eared fox | 0.20 | 0.11 | | - |
| Black wildebeest | 0.44 | 1.00 | | 0.42 |
| Black footed cat | - | 0.35 | | - |
| Bushpig | - | 1.00 | | - |
| Blesbok | 1.00 | - | | 0.02* |
| Buffalo | - | - | | 0.27 |
| Bubbling kassina | 1.00 | - | | - |
| Cape crow | 0.43 | - | | 0.44 |
| Cape fox | 0.20 | 0.01* | | 0.23 |
| Cape grysbok | 1.00 | - | | 0.17 |
| Cape hare | 0.02* | 0.93 | | 0.08 |
| Cape river frog | 0.66 | - | | 0.45 |
| Cape rock hyrax | 1.00 | - | | - |
| Cape legless skink | - | - | | 0.44 |
| Chicken | - | 1.00 | | - |
| Cape short-eared gerbil | - | | - | 0.03* |
| Cape springhare | - | 0.47 | | 0.63 |
| Caracal | 0.35 | - | | - |
| Cattle | 0.41 | 0.75 | | 0.71 |
| Chacma baboon | 0.00* | - | | 1.00 |
| Common duiker | 0.04* | 0.44 | | 0.53 |
| Common house martin | 0.43 | - | | 1.00 |
| Common mole-rat | 0.37 | 1.00 | | 0.57 |
| Desert pygmy mouse | 0.04* | 0.35 | | 0.65 |
| Eland | - | - | | 0.13 |
| Gemsbok | 0.17 | - | | 0.01* |
| Goat | 0.54 | 1.00 | | 0.26 |
| Greater kudu | 0.29 | 1.00 | | 0.04* |
| Jackal buzzard | - | 0.35 | | - |
| Ground squirrel | - | - | | 1.00 |
| Hairy-footed gerbil | 1.00 | - | | 0.25 |
| Helmeted guineafowl | 0.91 | - | | 0.53 |
| House mouse | - | - | | 1.00 |
| Karoo bush rat | 0.82 | 0.34 | | 0.04* |
| Laminate vlei rat | 0.83 | 0.32 | | 0.70 |
| Leopard | - | 1.00 | | 0.70 |
| Leopard tortoise | 1.00 | 0.65 | | 0.70 |
| Lion | - | - | | 0.27 |
| Meerkat | - | 0.35 | | - |
| Mountain reedbuck | 0.14 | - | | - |
| Namaqua rock rat | 0.81 | 0.07 | | 0.01* |
| Natal red rock hare | 0.69 | 0.47 | | - |
| Ostrich | - | 0.12 | | - |
| Pig | - | 0.20 | | 0.10 |
| Roan antelope | 0.24 | - | | - |
| Round-eared elephant shrew | **-** | 0.65 | | - |
| Serval | 0.09 | - | | - |
| Sheep | 0.15 | 0.11 | | - |
| Single-striped grass mouse | 0.29 | 0.51 | | 0.01* |
| Slender mongoose | 1.00 | - | | - |
| Small-spotted genet | 0.80 | 0.84 | | 0.53 |
| Soricidae | 0.06 | 0.11 | | 0.35 |
| Southern African vlei rat | 1.00 | 0.02* | | 0.10 |
| Southern African pygmy mouse | 0.02* | 1.00 | | 0.00* |
| Southern multimammate mouse | 0.95 | - | | 0.02* |
| Springbok | 0.24 | 0.29 | | 0.06 |
| Steenbok | 0.27 | 0.00* | | 0.02* |
| Vervet monkey | 0.52 | 0.35 | | 0.17 |
| Warthog | 0.09 | 0.50 | | 0.01* |
| Waterbuck | 0.83 | - | | - |
| Western rock elephant shrew | 0.66 | - | | - |
| White rhino | 0.20 | - | | - |
| Burchell’s zebra | 0.22 | - | | 0.06 |
| Wabler | - | - | | 0.25 |
| Mannikin | - | - | | 0.44 |
| Namibian long-eared bat | - | | - | 0.70 |
| Red-faced mousebird | - | - | | 0.44 |
| Large eared free-tailed bat | - | | - | 0.70 |
